# Evaluation of Intestinal Permeability and Liver Bacterial Translocation in Two Modern Broilers and Their Jungle Fowl Ancestor

**DOI:** 10.3389/fgene.2019.00480

**Published:** 2019-05-21

**Authors:** Mikayla F. A. Baxter, Sami Dridi, Dawn A. Koltes, Juan D. Latorre, Walter G. Bottje, Elizabeth S. Greene, Stephen W. Bickler, Jae H. Kim, Ruben Merino-Guzman, Xochitl Hernandez-Velasco, Nicholas B. Anthony, Billy M. Hargis, Guillermo Tellez-Isaias

**Affiliations:** ^1^Department of Poultry Science, University of Arkansas, Fayetteville, AR, United States; ^2^Department of Animal Science, Iowa State University, Ames, IA, United States; ^3^Division of Pediatric Surgery, Rady Children’s Hospital-University of California, San Diego, San Diego, CA, United States; ^4^Division of Neonatology, University of California, San Diego, San Diego, CA, United States; ^5^College of Veterinary Medicine, National Autonomous University of Mexico, Mexico City, Mexico

**Keywords:** chicken lines, nutritional rehabilitation, intestinal paracellular permeability, tight junctions, gut barrier function

## Abstract

The objective of this study was to evaluate the of intestinal permeability and liver bacterial translocation (BT) across a modern commercial broiler, a commercial broiler of 1995 genetics, and an unselected Jungle Fowl line. Modern 2015 (MB2015) broiler chicken, random bred line initiated from 1995 (RB1995), and the Giant Jungle fowl (JF). Chickens were randomly allocated to four different dietary treatments. Dietary treatments were (1) a control corn-based diet throughout the trial [corn-corn (C-C)]; (2) an early phase malnutrition diet where chicks received a rye-based diet for 10 days, and then switched to the control diet [rye-corn (R-C)]; (3) a malnutrition rye-diet that was fed throughout the trial [rye-rye (R-R)]; and (4) a late phase malnutrition diet where chicks received the control diet for 10 days, and then switched to the rye diet for the last phase [corn-rye (C-R)]. Paracellular permeability was evaluated using fluorescein isothiocyanate dextran (FITC-D). Liver BT was also evaluated. MB2015 and RB1995 consuming the rye-based diet showed increase serum levels of FITC-D when compared to the corn-fed chickens (*P* < 0.05). Overall, MB2015 appeared to have higher enteric permeability than the JF. To our knowledge, this would be the first paper to evaluate the effect of compensatory growth on intestinal permeability and liver BT. Further studies to evaluate microbiome and inflammatory markers in these chicken models are currently being evaluated.

## Introduction

Utilizing rye as a source of energy in poultry diets provides a unique model to induce intestinal inflammation due to the high non-starch polysaccharides (NSP) content and increase intestinal viscosity. This diet not only interferes with the absorption of nutrients reducing the growth of the birds but also causes alterations of the microbiota and luminal pH, poor bone mineralization, increased intestinal permeability, liver bacterial translocation (BT) and proliferation of *Clostridium* spp. (Choct et al., 2010; Tellez et al., 2014, 2015; Latorre et al., 2015, 2016). Our laboratory has recently optimized the use of fluorescein isothiocyanate-dextran (FITC-D), a 3–5 kDa fluorescent marker gavaged to birds and then measured in the serum as an indicator of paracellular transport in broiler chickens (Vicuña et al., 2015; Baxter et al., 2017; Latorre et al., 2018). The objective of this study was to evaluate the of intestinal permeability and liver BT across a modern commercial broiler, a commercial broiler of 1995 genetics, and an unselected Jungle Fowl line, using a compensatory model previously published (Baxter et al., 2018).

## Materials and Methods

### Animals, Diets and Experimental Design

All animal procedures were approved and in compliance with Institutional Animal Care and Use Committee (IACUC) at the University of Arkansas, Fayetteville (protocol #15006). Three lines of chickens were included in this study. For the modern broiler chickens (MB2015), 100 and 161-day-old mixed broiler chicks, Cobb-Vantress, (Siloam Springs, AR, United States) were used (*n* = 40 chickens/group). For the 1995 broiler chickens, 100 and 121-day-old mixed broiler chicks, from the random-bred line initiated from 1995 (RB1995) Cobb broiler chicken line (Harford et al., 2014) were used (*n* = 28 chickens/group). Moreover, for the Jungle fowl chickens (JF), 100 and 161-day-old mixed Giant Jungle fowl (Gyles et al., 1967) were used (*n* = 40 chickens/group). The reduced number of 1995 broilers utilized in this study was due to the low number of eggs produced by the breeding flock and a low hatch rate of the chicks. On the day of hatch, chicks were neck-tagged, weighed and randomly allocated to one of four dietary treatment groups in floor pens containing new pine shavings in a climate controlled room. Temperature and humidity were regulated to meet Cob Vantress management guidelines (Bourne, 2012). Chicks were maintained at 33°C with 30–50% relative humidity from 0 to 7 days of age. From 7 to 14 days of age, the temperature was reduced to 30°C with the relative humidity ranging from 40 to 60%. From 14 to 20 days of age, the temperature was reduced to 27°C, and relative humidity was maintained at 40–60%. The primary energy source of each diet was either corn or rye plus soybean meal to meet the recommended requirements of a broiler starter diet according to the (National Research Council, 1994). Table 1 lists the diet composition and nutrient content for each experimental diet. Both diets were formulated to be isonitrogenous. The dietary treatment was split into two phases, the first phase was from the day of hatch to day 10 and the second phase was day 10 to day 20. Dietary treatments were (1) a control diet where chickens were maintained on a corn-based diet throughout the trial (corn-corn, C-C); (2) an early phase malnutrition diet where chickens were on a rye-based diet for 10 d, and then switched to the control diet (rye-corn, R-C); (3) a malnutrition rye-diet that was fed throughout the trial (rye-rye, R-R); and (4) a late phase malnutrition diet where chickens received the control diet for 10 days, and then switched the rye diet for the last phase (corn-rye, C-R). Individual body weights were recorded at day 9 and 19 to determine appropriate dosing for FITC-D. On 10 days of age, fourteen chickens from each genetic line and on each treatment were randomly selected and euthanized via carbon dioxide asphyxiation, and blood (*n* = 14) and liver (*n* = 12) were collected. On 20 days of age, the remainder of the chickens were euthanized, and blood and (*n* = 14) and liver (*n* = 12) were collected from each treatment in each genetic line evaluated.

**TABLE 1:** Composition and nutrient content of the experimental diets (%).

Item	Rye-based diet	Corn-based diet
**Ingredient**		
Corn	–	57.32
Rye	58.27	–
Soybean meal	31.16	34.66
Poultry fat	6.30	3.45
Dicalcium phosphate	1.80	1.86
Calcium carbonate	1.10	0.99
Salt	0.38	0.38
DL-Methionine	0.35	0.33
Vitamin premix^1^	0.10	0.20
L-Lysine hydrochloride	0.22	0.31
Choline chloride 60%	0.10	0.20
Mineral premix^2^	0.12	0.12
Threonine	0.08	0.16
Antioxidant^3^	0.02	0.02
**Calculated analysis**		
Metabolizable energy (kcal/kg)	2850	3035
Crude protein, %	22.38	22.16
Lysine, %	1.32	1.35
Methionine, %	0.64	0.64
Methionine + cystine, %	0.98	0.99
Threonine, %	0.86	0.91
Tryptophan, %	0.30	0.28
Total calcium, %	0.90	0.9
Available phosphorus (%)	0.45	0.45
Sodium (%)	0.16	0.16

*^1^Vitamin premix supplied the following per kg: vitamin A, 20,000 IU; vitamin D3, 6,000 IU; vitamin E, 75 IU; vitamin K3, 6.0 mg; thiamine, 3.0 mg; riboflavin, 8.0 mg; pantothenic acid, 18 mg; niacin, 60 mg; pyridoxine, 5 mg; folic acid, 2 mg; biotin, 0.2 mg; cyanocobalamin, 16 μg; and ascorbic acid, 200 mg (Nutra Blend LLC, Neosho, MO, United States). ^2^Mineral premix supplied the following per kg: manganese, 120 mg; zinc, 100 mg; iron, 120 mg; copper, 10 to 15 mg; iodine, 0.7 mg; selenium, 0.4 mg; and cobalt, 0.2 mg (Nutra Blend LLC, Neosho, MO, United States). ^3^Ethoxyquin.*

### Liver Bacterial Translocation

The right liver was aseptically removed from each chicken, collected into VWR^®^ sterile sampling bags (VWR, Radnor, PA, United States), homogenized and weighed as previously described by Tellez et al. (2014, 2015) and Latorre et al. (2015). Samples were then diluted 1:4 based on tissue weight with sterile 0.9% saline. Liver samples (*n* = 12/chicken line) were then transferred to sterile 96 well Bacti flat bottom plate and diluted tenfold before being plated on TSA. Samples were incubated under aerobic conditions at 37°C for 24 h.

### Serum Determination of FITC-D

Baxter et al. (2017) have previously described the FITC-D protocol. In brief, chickens were orally gavaged with FITC-D (8.32 mg/kg) dissolved in non-sterile milliQ water 1 h before sample collection, chickens were euthanized and blood was collected from the femoral vein (*n* = 14/chicken line) in 13 mm × 100 mm disposable borosilicate glass culture tubes (VWR, Radnor, PA, United States) and allow to clot for 4 h at room temperature. Serum was isolated from the blood by centrifuging tubes at 2000 × *g* for 10 min at 4°C. Serum was then removed and diluted 1:5 in sterile 0.9% saline to a total volume of 100 μl in 96 well flat bottom black plate. FITC-D was measured at an excitation wavelength of 485 nm and an emission wavelength of 528 nm (Synergy HT, Multi-mode microplate reader, BioTek Instruments, Inc., VT, United States). Serum fluorescent concentrations were then determined using a standard curve and sera of chickens not given FITC-D.

### Statistical Analysis

Data were analyzed within treatment using a linear mixed model procedure in SAS (PROC MIXED; SAS Institute Inc., 2002). The model fit dietary treatment, genetic line, and the interaction between the dietary treatment and genetic line as fixed effects for all variables. Significance was set at *P* < 0.05. When model effects were significant, pairwise comparisons were performed in SAS using the pdiff statement and corrected for multiple tests using Tukey *post hoc* adjustment.

## Results and Discussion

The results of the evaluation of a nutritional rehabilitation model on serum FITC-D in three genetic chicken lines fed rye or corn at different time points are summarized in Table 2. At 10 days of age, chickens that received a rye-based diet in both the MB2015 and the RB1995 genetic lines showed a significant increase in serum FITC-D concentration when compared with the chickens that received the corn-based diet. However, diet did not affect gut permeability in JF chickens. Interestingly and regardless of the dietary treatment, serum FITC-D levels varied significantly within genetic lines with MB2015 had the highest levels of serum FITC-D, followed by RB1995, and JF had the lowest levels (Table 2). The chicken intestinal tract is primarily developed by 3 weeks of age and includes barrier function. Furthermore, the lack of genetic selection on the jungle fowl makes it likely that it takes these chickens longer to have a fully functioning intestinal tract. Therefore, the higher levels of serum FITC-D at 10 days of age may be due to the immaturity of the intestinal tract due to age or genetic selection. At 20 days of age, chickens in the R-C treatment group had the same level of serum FITC-D as those maintained on a rye-based diet in both MB2015 and RB1995 lines. Also, the C-R treatment group had a significantly higher level of serum FITC-D than the R-C group. However, chickens maintained on the corn-based diet had a significantly lower serum FITC-D than those chickens on the R-C treatment group. This phenomenon was observed in both broiler genetic lines (MB2015 and RB1995). In contrast, there were no significant differences observed in gut barrier permeability in JF chickens regardless of the dietary treatment. Another impressive result was that JF chickens that received a corn-based diet, for 20 days, showed significantly higher levels of FITC-D when compared with the MB2015 and RB1995. Nevertheless, the opposite effect was observed in MB2015 and RB1995 chickens’ in the C-R treatment group, which had significantly higher levels of serum levels of FITC-D when compared with JF chickens. No significant interaction between genetic lines was observed in chickens in the R-R and R-C treatment groups. Regardless of the genetic line, treatment had a significant effect on gut permeability where corn-fed chickens had significantly lower serum FITC-D than those chickens fed rye at any phase of the experiment. The genetic line also had a significant effect on gut permeability where modern broiler had significantly higher serum FITC-D levels than 1995 broilers and JF (Table 2). On the other hand, at 10 days of age, chickens from the MB2015 and RB1995 on the corn-based diet had a significant increase in liver BT when compared with the JF line. However, there were no significant differences between dietary treatments within each of the genetic lines (Table 2). Regardless of genetic lines, rye fed chickens tended to have higher liver BT than corn-fed chickens. At 20 days of age, MB2015 and RB1995 chickens consuming a rye-based diet in the second phase of the experiment had higher liver BT compared to those chickens fed a corn-based diet. Similar results were observed in the serum FITC-D levels, and the C-R treatment group had a significantly higher liver BT than the R-C group in both MB2015 and RB1995 chickens. In contrast, there was no significant difference between dietary treatments in JF. For chickens in the R-R treatment group, MB2015 had significantly higher liver BT than the JF (Table 2). Regardless of the genetic line, treatment had a significant effect on hepatic BT, chickens fed corn in the second phase of the experiment had significantly lower liver BT than the chickens fed rye-based diet. Regardless of dietary treatment, 1995 broiler had significantly higher liver BT than the jungle fowl. Interestingly, in both modern broiler lines, chickens in the group R-C showed an improvement in gut barrier function when compared with R-R or C-R chickens, suggesting that the intestinal epithelium recovered some of its function. While the restoration of liver BT is restored when fed a corn-based diet for 10 days, serum FITC-D levels remain elevated in broilers that received the R-C diet compared to broilers on the C-C diet. The chicken intestinal tract is primarily developed by 3 weeks of age and includes barrier function. Furthermore, the lack of genetic selection on the jungle fowl makes it likely that it takes these chickens longer to have a fully functioning intestinal tract. Therefore, the higher levels of serum FITC-D at 10 days of age may be due to the immaturity of the intestinal tract due to age or genetic selection.

**TABLE 2:** Evaluation of intestinal permeability and liver bacterial translocation in three genetic chicken lines fed rye or corn at varying time points.

Day of evaluation	Treatment	MB2015	Genetic line RB1995	Jungle fowl	Variable	*P*-value
**Serum FITC-D (ng/mL)**						
10 days	Corn	1162.8 ± 107.6^b, x^	217.0 ± 60.1^b, y^	39.8 ± 18.0^a, z^	trt	<0.0001
	Rye	1738.7 ± 124.1^a, x^	642.2 ± 108.1^a, y^	51.6 ± 17.6^a, z^	line	<0.0001
					trt*line	<0.0001
20 days	Corn-Corn	8.5 ± 8.5^c, z^	8.1 ± 8.5^c, z^	126.6 ± 38.3^b, y^	trt	<0.0001
	Rye-Corn	158.3 ± 26.8^b, z^	253.5 ± 27.5^b, z^	184.3 ± 31.7^ab, z^	line	0.0894
	Rye-Rye	221.0 ± 64.0^b, z^	386.1 ± 37.8^a, z^	265.8 ± 32.6^a, z^	trt*line	<0.0001
	Corn-Rye	433.1 ± 47.0^a, y^	413.4 ± 46.9^a, y^	257.6 ± 25.7^a, z^		
**Liver bacterial translocation (Log_10_ cfu/g)**						
10 days	Corn	2.0 ± 0.4^a, y^	1.8 ± 0.3^a, y^	0.8 ± 0.3^a, z^	trt	0.0589
	Rye	2.4 ± 0.4^a, z^	2.1 ± 0.3^a, z^	1.5 ± 0.3^a, z^	line	0.001
					trt*line	0.7922
20 days	Corn-Corn	0.4 ± 0.3^a, z^	0.9 ± 0.3^a, z^	0.6 ± 0.3^a, z^		
	Rye-Corn	0.4 ± 0.3^a, z^	1.1 ± 0.3^a, z^	1.1 ± 0.3^a, z^	trt	<0.0001
	Rye-Rye	2.7 ± 0.4^b, y^	2.4 ± 0.3^b, yz^	0.9 ± 0.4^a, z^	line	0.0307
	Corn-Rye	2.1 ± 0.3^b, z^	2.0 ± 0.3^b, z^	1.1 ± 0.3^a, z^	trt*line	0.0042

*Data are expressed as the mean ± SE. (*P* < 0.05). ^a–c^Indicates significant differences between the treatments within the column at each time point, respectively. ^x–z^Indicates a significant difference between the genetic lines within the rows at each time point, respectively.*

The gut barrier is a complex, multicomponent, interactive, and bidirectional entity. Intestinal permeability reflects just one function of the barrier (Quigley, 2016). However, even when disrupted, the paracellular pathway is not capable of transporting large molecules or whole bacteria (Hooks and O’Malley, 2017). Hence, the intestinal barrier and permeability are not interchangeable terms (Azpiroz et al., 2004). Even though is not clear how microbes of 0.5 to 1 μm gain access to epithelial soma through densely packed microvilli, recent studies suggest that interferon-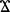 and myosin light chain kinase are involved in bacterial endocytosis (Wu et al., 2014). Transcytotic commensal or pathogenic penetration may contribute to chronic systemic inflammation (Wiest and Garcia-Tsao, 2005; Odenwald and Turner, 2013). Since the majority of the venous blood from the intestinal tract is drained into the portal circulation, the liver is the first organ to encounter nutrients or gut-derived bacteria (Berg and Garlington, 1979; Greenwood-Van Meerveld, 2012). Genetic selection for growth has caused vastly different growth rates between different strains of birds. The modern broiler has been heavily selected for growth, while the 1995 broiler line has been moderately selected for growth. Both genetic lines were selected from a common ancestor not selected for growth, the Jungle Fowl (Wall and Anthony, 1995; Harford et al., 2014). To our knowledge, this would be the first paper to evaluate the effect of compensatory growth on intestinal permeability and liver BT. Further studies to evaluate the role of IFN- 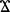 and transcytosis of commensal bacteria and microbiota analysis using this nutritional rehabilitation model are currently being evaluated.

## Ethics Statement

All animal procedures were approved and in compliance with Institutional Animal Care and Use Committee (IACUC) at the University of Arkansas, Fayetteville (protocol #15006).

## Author Contributions

MB, SD, DK, and GT-I contributed to the overall study design and supervised to all the research. JL, WB, EG, SB, JK, and RM-G carried out the experiments and acquired of the data. MB and GT-I drafted and revised the first version of the manuscript and analyzed the data. NA, WB, BH, and GT-I drafted the article and revised it critically for important intellectual content. MB, XH-V, and GT-I were responsible for the final editing of the manuscript. All authors reviewed and finally approved the manuscript.

## Conflict of Interest Statement

The authors declare that the research was conducted in the absence of any commercial or financial relationships that could be construed as a potential conflict of interest.
